# BANF1 is a novel prognostic biomarker linked to immune infiltration in head and neck squamous cell carcinoma

**DOI:** 10.3389/fimmu.2024.1465348

**Published:** 2024-10-08

**Authors:** Yaodong He, Huan Li, Jing Li, Junhong Huang, Rong Liu, Yanbing Yao, Yating Hu, Xinjie Yang, Jianhua Wei

**Affiliations:** State Key Laboratory of Oral & Maxillofacial Reconstruction and Regeneration, National Clinical Research Center for Oral Diseases, Shaanxi Clinical Research Center for Oral Diseases, Department of Oral and Maxillofacial Surgery, School of Stomatology, Fourth Military Medical University, Xi’an, China

**Keywords:** BANF1, TCGA, immune infiltration, head and neck squamous cell carcinoma, prognostic biomarker

## Abstract

**Background:**

Barrier-to-autointegration factor 1 (BANF1) is an abundant and ubiquitously expressed postnatal mammalian protein that is overexpressed in numerous human cancers and can promote cancer cell proliferation. However, the role of BANF1 in prognosis remains unclear in head and neck squamous cell carcinoma (HNSCC).

**Methods:**

BANF1 expression data were obtained from the GEO and TCGA databases. We used Cox regression and Kaplan–Meier curves to assess the prognostic potential of BANF1. The role of BANF1-related genes was investigated using Kyoto Encyclopedia of Genes and Genomes (KEGG) and Gene Ontology (GO) enrichment analyses. In addition, we explored the link between BANF1, drug sensitivity, and the tumor immune microenvironment. Finally, functional *in vitro* and *in vivo* assays were used to explore the effects of BANF1 on tumor growth and metastasis of HNSCC.

**Results:**

BANF1 was markedly overexpressed in HNSCC and was correlated with clinicopathological characteristics. According to survival analysis, BANF1 can be inversely correlated with patient survival and can act as a prognostic risk indicator. IC50 values for chemotherapeutic treatments indicated that the group with high BANF1 expression was more responsive to most antitumor treatments. Furthermore, higher TIDE scores were observed in the low BANF1 expression group, indicating a decline in the efficacy of immune checkpoint inhibitor therapy. Functionally, the malignant biological behavior of HNSCC cell lines was inhibited when BANF1 expression was knocked down.

**Conclusion:**

BANF1 can promote tumor progression in patients with HNSCC. BANF1 shows great promise as a potential biomarker to assess the prognosis.

## Introduction

Head and neck squamous cell carcinoma (HNSCC) is responsible for almost 90% of head and neck malignancies (1). HNSCC occurs primarily in the oral cavity, nasal cavity, sinuses, pharynx, and larynx (2–4). HNSCC has an annual diagnosis rate of about 830,000 cases, which represents 8% of all cancer diagnoses. This tumor exhibits a high level of aggressiveness, resulting in a death rate of up to 50% in a period of 5 years (5, 6). HNSCC is a diverse collection of malignancies that exhibit significant differences in tumor site, histological subtype, molecular characteristics, and prognosis (7, 8). HNSCC is influenced by various risk factors, such as tobacco use, alcohol consumption, exposure to environmental pollutants, and viral infections, including human papillomavirus (HPV) and human herpesvirus (HHV) (9). These variables can independently or in combination influence the risk of HNSCC and may vary depending on the specific location of the tumor. Treatment selection is influenced by various factors, including TNM staging, overall patient health, tumor size, nutritional status, availability of resources, and prognosis (10). Immunotherapeutic approaches have advanced significantly in recent years. These include monoclonal antibodies, vaccinations, immunomodulatory antibodies, lysogenic viruses, and T-cell transplants (11). These techniques are routinely utilized in clinical practice. Although humans have made considerable advances in the treatment of HNSCC, patient prognosis has not shown a significant improvement. The overall 5-year survival rate for HNSCC remains poor, ranging from 40% to 50% (12). The main obstacle to HNSCC treatment is the significant recurrence rate and/or metastasis in patients. This not only highlights the difficulties in treating HNSCC, but also uncovers the intricate molecular mechanisms involved in the genesis and progression of cancer. Therefore, it is imperative to investigate potent systemic treatments by obtaining a more thorough understanding of the molecular pathogenesis underlying HNSCC. Furthermore, it is crucial to pinpoint new therapeutic targets related to the prognosis of HNSCC and infiltration of the immune system.

Barrier to autointegration factor 1 (BAF), which is encoded by the BANF1 gene, is sometimes referred to as NGPS or BCRP1. It is located on chromosome 11q13.1. Originally identified and named for its function in binding to viral cytoplasmic DNA, the highly organized control of BAF in conjunction with multiple binding partners and its ability to attach and compact DNA are crucial for important cellular functions. These include the formation of the nuclear membrane after cell division, repairing damage to the protective barrier of the nuclear envelope, regulating gene expression, and responding to DNA damage (13–17). BAF dimers bind LAP2-Emerin-MAN1 (LEM) structural domain proteins and A-type lamellipodia proteins to the nuclear envelope (NE) by attaching to the LEM structural domains of proteins to form Ig-like folds on A-type lamellipodia proteins (18). Each individual BAF monomer has the ability to connect to DNA in a manner that is independent of the specific DNA sequence. This allows the formation of bridges between DNA strands and facilitates functional cross-linking (56). The capacity of BAF to establish DNA-protein complexes is crucial for BAF to perform its primary biological function. DNA binding by BAF in cells is controlled by phosphorylation at the N-terminal (19). BAF can both enhance and inhibit the expression of genes within an organism; BAF collaborates with other gene regulators to selectively influence the expression of specific genes (20). Multiple studies have demonstrated that BANF1 is related to the growth, infiltration, and spread of various tumor cells, such as in gastric (21), liver (22), breast (23), esophageal (24), and cervical malignancies (25). Furthermore, a correlation between BANF1 and the prognosis of certain types of cancer has been shown (26). Wang et al. (27) discovered that BANF1, which originates from tumors, plays a crucial role in the immune response of the body against tumors. Knockdown of BANF1 expression in cancer cells genetically induces activation of innate immunity through the cGAS/STING pathway. This activation results in the production of several interferon-stimulated genes (ISGs) and inflammatory chemokines, which attract CD8+T cells to the tumor microenvironment (TME). Furthermore, simultaneous elimination of BANF1 and the administration of anti-PD-1 antibodies significantly improves the effectiveness of antitumor treatment. Based on the findings of Xu et al. (28), BANF1 is highly expressed in gastric cancer and facilitates proliferation and multiplication of gastric cancer cells. Zhang et al. (57) demonstrated that BANF1 expression was increased in breast cancer and was associated with the spread of cancer cells to the lymph nodes. Sandoval et al. (29) discovered that there are specific associations between ERG and ATP-dependent mammalian SWI/SNF (BAF) chromatin remodeling complexes. These connections are crucial for the ERG-mediated base-to-lumen transition, which is necessary for the targeted action of BAF complexes, gene expression, prostate cancer cell growth, and the overall base-to-lumen transition driven by ERG. Therefore, it is logical to propose that BANF1 may have a significant impact on the HNSCC pathogenesis. However, the exact mechanism by which BANF1 influences HNSCC is not fully understood, and further research is necessary to explore the connection between BANF1 and relevant genes in HNSCC.

The present study examined BANF1 expression levels in HNSCC using data from databases available to the public. We evaluated the relationships between clinicopathological characteristics, overall survival (OS), and BANF1 expression. The relationship between tumor immune cell infiltration and BANF1 expression were also examined. Lastly, we investigated whether BANF1 affects tumor growth and metastasis in HNSCC. Our findings support the involvement of BANF1 in the carcinogenesis and prognosis of HNSCC and may point to a possible biomarker for the prognosis and treatment of the disease.

## Materials and methods

### BANF1 expression analysis

A total of 548 patients with HNSCC and 44 cases with available paraneoplastic tissue, gene expression data, and corresponding clinical data were recovered from TCGA database. GSE30784, GSE23558, and GSE37991 datasets were selected and downloaded from GEO. The association of the BANF1 RNA expression profile with different tumor pathologic stages in humans was explored using UALCAN (http://ualcan.path.uab.edu/analysis.html).

### Evaluation of prognostic value

Survival analyses were performed by the Kaplan–Meier (KM) method using the median BANF1 expression as a threshold. We used both univariate and multivariate Cox regression analyses to determine whether BANF1 may be utilized as a prognostic predictor.

### Analysis of BANF1 co-expression and functional enrichment

The biological involvement of BANF1 in HNSCC was investigated by comparing high and low gene expression groups using differential expression gene (DEG) analyses, and |logFC|> 1 and FDR <0.05 were used as parameters for significant DEGs (**Supplementary Table 1**). Gene Set Enrichment Analysis (GSEA) was used to explore BANF1-related pathways and phenotypes and to compare biological functions between patients with high and low BANF1 expression. Gene Ontology (GO) and Kyoto Encyclopedia of Genes and Genomes (KEGG) gene sets were obtained from authorized portals. The “c5.go.v2022.1.Hs.symbols.gmt” and “c2.cp.reactome. v2022.1.Hs.symbols.gmt” subsets were used to assess oncogenic or tumor-associated pathways in HNSCC and to perform functional analyses using the limma, clusterProfiler, and GSEA software packages in R.

### Single-cell RNA sequencing analysis

We explored BANF1 expression at the single-cell level in three HNSCC single-cell sequencing datasets (GSE103322, GSE139324 and GSE172577) using the TISCH database.

### Immune infiltration analysis

A detailed analysis of immune cell type infiltration involved extracting BANF1 gene expression data from each sample from TCGA HNSCC dataset. Using modules such as QUANTISEQ, TIMER, and XCELL, we explored the correlation between immune cell infiltration and BANF1 expression in HNSCC. TME assessment using the R package ESTIMATE enabled the calculation of stromal and immune scores for each patient based on their gene expression profile.

### Immune checkpoint inhibitor therapy and gene expression

The Tumor Immune Dysfunction and Exclusion (TIDE) Platform has greatly improved our understanding of TME by offering a streamlined approach to forecast the potential efficacy of immune checkpoint inhibitors (ICIs) (30). We used the Imvigor210 data set to predict the impact of immunotherapy on the two distinct groups of patients with HNSCC according to their BANF1 expression levels: high and low. Patients with a high TIDE score were more likely to have immunological rejection, suggesting a reduced probability of benefiting from immunotherapy. We utilized the TIDE database to evaluate the prognostic efficacy of BANF1 and other biomarkers in HNSCC immunotherapy groups. The performance of a marker was considered better when its area under the curve (AUC) value is higher.

### Analysis of chemotherapeutic sensitivity

The Genomics of Drug Sensitivity in Cancer (GDSC), a public pharmacogenomics database, was used to assess and predict chemotherapy responses in patients with HNSCC belonging to various risk groups in the TCGA database (31). The study examined the half-maximum inhibitory concentration (IC50), which is a reliable indicator of sensitivity to chemotherapy, between two groups: high-risk and low-risk. A statistically significant difference was determined if the p-value was less than 0.05. CellMiner (http://discover.nci.nih.gov/cellminer/) identified a correlation between BANF1 expression and drug response.

### Cell lines

Human normal oral epithelial cells (HOK) were obtained from Wuhan Pricella Biotechnology Co., Ltd., while human HNSCC cell lines, HN4, HN6, SCC9, and CAL27, were obtained from the Typical Cultures Preservation Committee (TCPC) Cell Bank of the Chinese Academy of Sciences (CAS), Shanghai, China. DMEM medium, 10% fetal bovine serum, and 100 U/mL penicillin were used for all cell cultures. Cells were cultured at 37°C with 5% CO2.

### RNA extraction and quantitative real-time polymerase reaction

Total RNA was extracted from cell lines using Thermo Fisher Scientific’s TRIzol reagent (USA). The NanoDrop spectrophotometer (Thermo Fisher Scientific, USA) was used to ascertain the quantity and purity of RNA. Prime Script RT Master Mix, manufactured by Takara (Cat. #RR047A) was used for the reverse transcription of all RNA and qRT-PCR was performed using CFX96 Touch Real-Time PCR Detection System (Bio-Rad) according to the manufacturer’s instructions. GAPDH was utilized for internal reference and the reaction was performed in a two-step process using the following conditions: initial predenaturation at a temperature of 95°C for a duration of 30 seconds, followed by denaturation at 95°C for 5 seconds, and annealing or extension at 60°C for 30 seconds. This cycle was repeated 50 times. The Ct method was employed to measure expression levels and the 2–ΔΔCt method was used to calculate relative gene expression.

### Lentiviral design and transfection

Anhui General Gene Technology Co., Ltd. Successfully designed and constructed targeted lentiviral vectors for gene silencing experiments using BANF1 the gene sequence. The sh-BANF1 sequence and negative control (sh-NC) were designed to achieve effective inhibition of target genes. In this study, GV248 lentiviral RNAi vectors with titers of 9×10^8^ TU/mL and 5×10^8^ TU/mL, respectively. Green fluorescent protein gene was integrated into the transfer plasmid to monitor successful cell transfection or not and to remove untransfected cells by puromycin selection to construct stable transfected strains. qRT-PCR was used to determine BANF1 mRNA expression in SCC9 and CAL27 cells from the sh-BANF1 group and the sh-NC group.

### Cell-counting Kit-8 proliferation assay

Transfected SCC9 and CAL27 cells were diluted to 30,000 cells/mL and 100 μL of the cell suspension was added to each well. Each group of cells was inoculated into four 96-well plates and cultured in the incubator for 0, 24, 48, or 72 h. Next, 10 μL of CCK-8 solution was added to each well, incubated in the incubator for 2 h, and then the absorbance at 450 nm was determined with an enzyme marker.

### Colony formation assay

The transfected SCC9 and CAL27 cells of each group were stained with Taipan blue for viable cell counting, and 1000 viable cells were seeded per well in a six-well plate, so that the number of clones formed in each well was between 50 and 200. Next, cells were incubated for 10 to 15 days, fix with paraformaldehyde, wash with PBS once, stained with crystal violet for 30 minutes, and washed with water, and finally allowed to air dry. Images of the cultures were captured, and the number of clones formed were counted. To ensure precision, each experiment was performed three times.

### Wound healing assay

Once achieving 80% cell confluence of transfected SCC9 and CAL27 cells seeded in 12-well plate, the monolayers were scraped with the tip of a 10 μL pipette. Following three washes with PBS to remove cellular debris, fresh medium containing serum was introduced. At the 0 and 24-h time points following scratching, three high-magnification fields were captured to acquire representative images of cell migration. The scratch width was determined using ImageJ.

### Transwell migration and invasion assays

A serum-free cell suspension was prepared, and the cell density was adjusted to 1×10^5^ cells/mL. A 100 µL volume of cell suspension was added to the desired number of chambers in a 24-well plate, and 600 µL of 30% serum medium was added to the lower chamber. After a 24-h incubation, cells were fixed with 4% paraformaldehyde for 15 minutes, washed once with PBS, stained with crystal violet for 10 minutes, and the chambers were immersed and rinsed several times. Three fields of view were randomly selected and the number of cells in the filter membranes were counted using a microscope; their average value was calculated. Three experiments are performed for each sample. For the invasion test, the Transwell system filters were coated with Matrigel (BD Biosciences), and the remaining processes were identical to those of the migration assay.

### Xenografts in mice

Immunodeficient nude mice, aged 4 weeks, were purchased from the Laboratory Animal Center of the Air Force Medical University. Breeding conditions and all operational procedures were conducted in accordance with the requirements of the Animal Ethics Committee of the Air Force Medical University. Twelve nude mice were equally and randomly divided into two sh-NC and sh-BANF1 groups. After stable transfection of SCC9 cells, they were cultured to the logarithmic growth stage and resuspended with PBS to reach a cell density of 5×10^6^ cells/mL, and then 200 μl of cell suspension was injected into the mice models. Once subcutaneous tumors formed, mice were examined every 3 days, their weight and tumor size are recorded. Mice were euthanized by cervical dislocation nearly 28 days after inoculation. Tumors samples are placed in a refrigerator at -80°C for future analysis and study.

### Statistical analysis

A Wilcoxon rank sum test was used to analyze BANF1 expression levels between HNSCC tissues and non-tumor tissues downloaded from the GEO database. Cox regression was performed using multivariate and univariate analyses. The TME score and IC50 were analyzed using the Wilcoxon test to compare the high- and low-expression groups. Furthermore, we estimated the correlation coefficients between BANF1 expression and immune-infiltrating cell scores by Pearson’s correlation analysis. All statistical analyses were performed using R v.4.1.1 software. Unless otherwise stated, P < 0.05 was considered statistically significant.

## Results

### High expression of BANF1 in HNSCC

**Figure 1:**
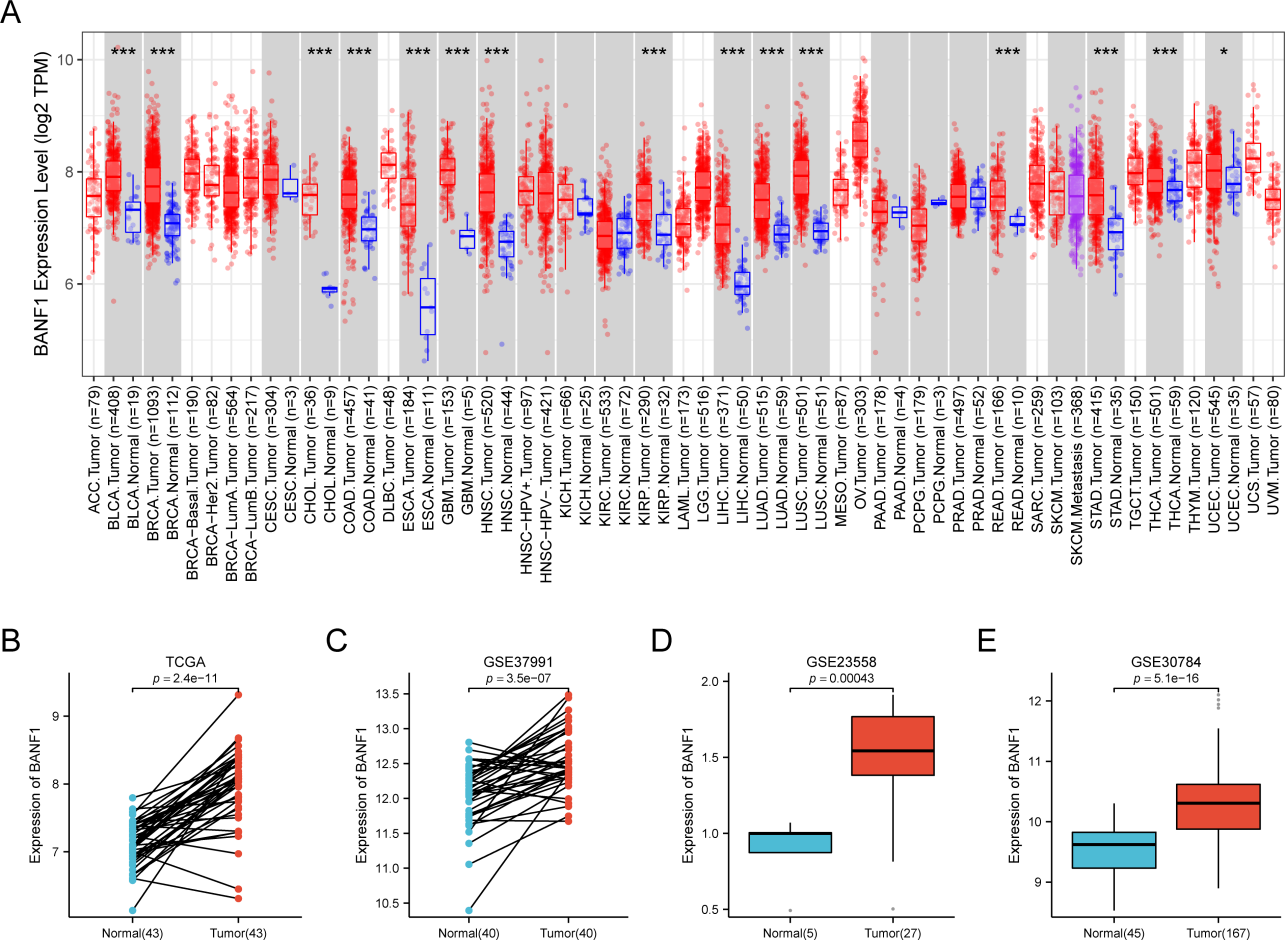
The expression of BANF1 was markedly elevated in HNSCC tissues. **(A)** The gene expression profiles of BANF1 in the pan-cancer dataset of the TCGA database. **(B)** In the TCGA database, the expression level of BANF1 was elevated in HNSCC tissue compared to the neighboring normal tissue. **(C–E)** The expression level of BANF1 was higher in tumor tissues in the GSE37991, GSE23558, and GSE30784 datasets. *P<0.05, **P<0.01, ***P<0.001.

Our comprehensive analysis, which incorporated data from the TIMER2 databases, revealed distinct and tumor-specific expression patterns of BANF1 in 33 different cancer tissues. The expression of BANF1 in 15 tumors was higher than the matched normal tissues, including BLCA, BRCA, CHOL, COAD, ESCA, GBM, HNSC, KIRP, LIHC, LUAD, LUSC, READ, STAD, THCA and UCEC (**Figure 1A**). To confirm the up-regulation of BANF1 expression in HNSCC, we performed a verification analysis using TCGA data sets. Based on the results acquired, BANF1 mRNA levels in tumor tissues were significantly higher than those of healthy tissues (**Figure 1B**). The increased expression of BANF1 in HNSCC tissues was verified using samples retrieved from GEO (accession numbers: GSE23558, GSE30784, and GSE37991 datasets) (**Figures 1C–E**).

**Figure 2:**
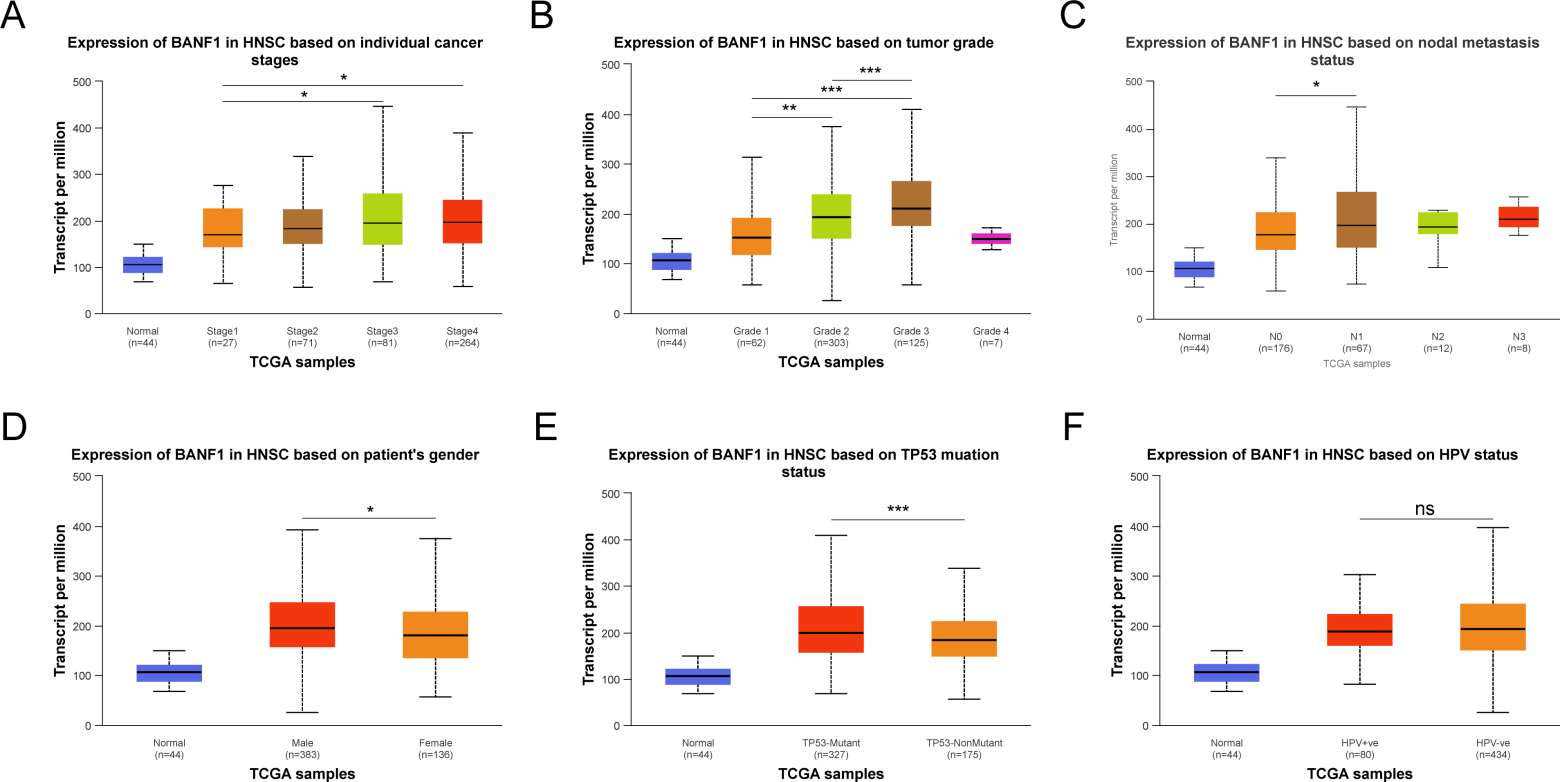
Comparison of BANF1 expression in different subgroups of Tumor stage **(A)**, Tumor grade **(B)**, Nodal metastasis status **(C)**, Gender **(D)**, TP53 status **(E)**, and HPV status **(F)**. *P<0.05, **P<0.01, ***P<0.001. ns indicated no significance.

The UNCLAN program was used to evaluate clinical subgroups based on variations in BANF1 expression between normal samples in patients with HNSCC. **Figures 2A–F** demonstrate a notable increase in BANF1 expression among various subgroups of patients with HNSCC, such as those with TP53 mutations, presence of metastasis, sex, and varying tumor grades and stages. This suggests that BANF1 could serve as a promising biomarker for patients with HNSCC. However, no variation in HPV infection status was observed.

### Prognostic value of BANF1 in HNSCC

**Figure 3:**
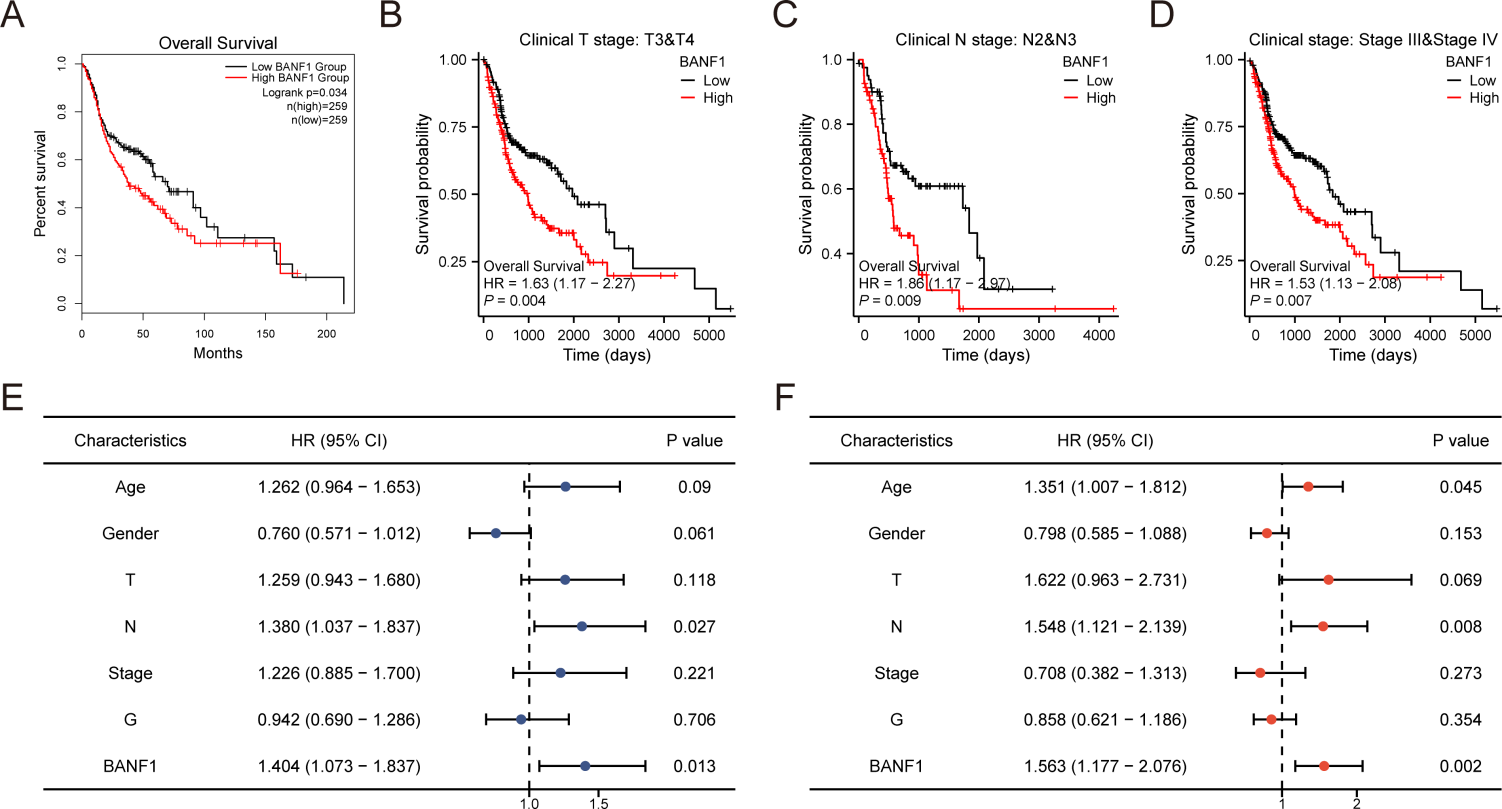
Investigation on the predictive importance of BANF1 in HNSCC. **(A–D)** Survival curves for patients with advanced HNSCC from the TCGA dataset were generated using the Kaplan-Meier plotter database. **(E, F)** Univariate and multivariate analyses were conducted to examine the relationship between overall survival and clinicopathologic features in individuals with HNSCC.

Kaplan–Meier plotter was used to investigate the effects of BANF1 on overall survival in patients with HNSCC. When BANF1 expression was high, patients with HNSCC had a worse prognosis (**Figure 3A**). The results indicated that BANF1 expression was associated with outcome in patients with advanced HNSCC (**Figures 3B–D**). In terms of OS, the results of univariate analysis showed that N-stage and high BANF1 expression (P = 0.027, P = 0.013, respectively) were correlated with the OS of patients, whereas age, sex, and pathological grade were not significantly correlated with the OS of patients (P > 0.05). Multifactorial analysis also determined that stage N and BANF1 expression (P = 0.008, P = 0.002, respectively) were independent risk factors for unfavorable OS in HNSCC (**Figures 3E, F**). These findings suggest that BANF1 expression is strongly associated with poor prognosis in HNSCC patients, highlighting its potential as a prognostic biomarker for this disease.

### Analysis of BANF1-related genes

**Figure 4:**
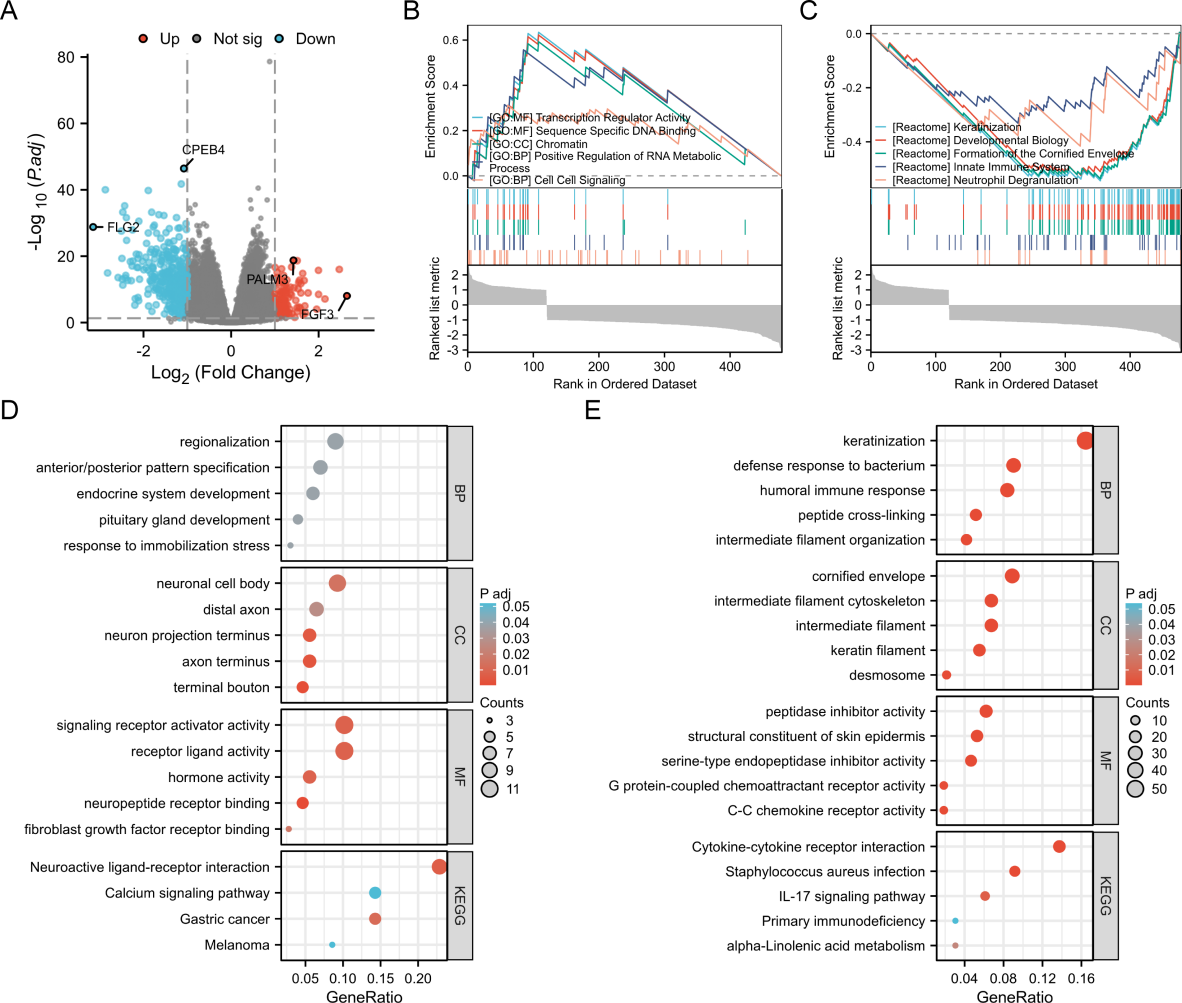
Functional enrichment analysis of BANF1 in HNSCC. **(A)** Volcano plot showing DEGs between high and low expressing BANF1 groups in HNSCC. **(B, C)** GSEA enrichment plot showing the correlation of high and low BANF1 expression with different tumor-related pathways. **(D, E)** GO and KEGG pathway analysis of up- and down-regulated DEGs in HNSCC.

A total of 488 DEGs were identified between the BANF1 high- and low-expression groups, of which 124 genes showed up-regulation and 364 genes showed down-regulation (**Figure 4A**). Furthermore, GSEA showed that keratinization, developmental biology, cornified envelope formation, innate immune system, and neutrophil degranulation were negatively regulated in patients with BANF1 expression. High BANF1 expression was associated with transcription regulator activity, sequence specific DNA binding, chromatin, positive regulation of RNA metabolic process, and cell-cell signaling (**Figures 4B, C**). We also performed GO and KEGG pathway analysis, which revealed that the humoral immune response, peptidase inhibitor activity, cytokine-cytokine receptor interaction, and the IL-17 signaling pathway were enriched in patients with down-regulated genes, however, up-regulated genes were predominantly enriched in regionalization, neuronal cell body, signaling receptor activator activity and neuroactive ligand-receptor interaction, and the calcium signaling pathway (**Figures 4D, E**). In conclusion, these gene enrichment studies suggest that BANF1 is important in the immune response of HNSCC, as well as in the invasion of cancer cells through the cell adhesion pathway.

### Relationship between BANF1 and tumor microenvironment

**Figure 5:**
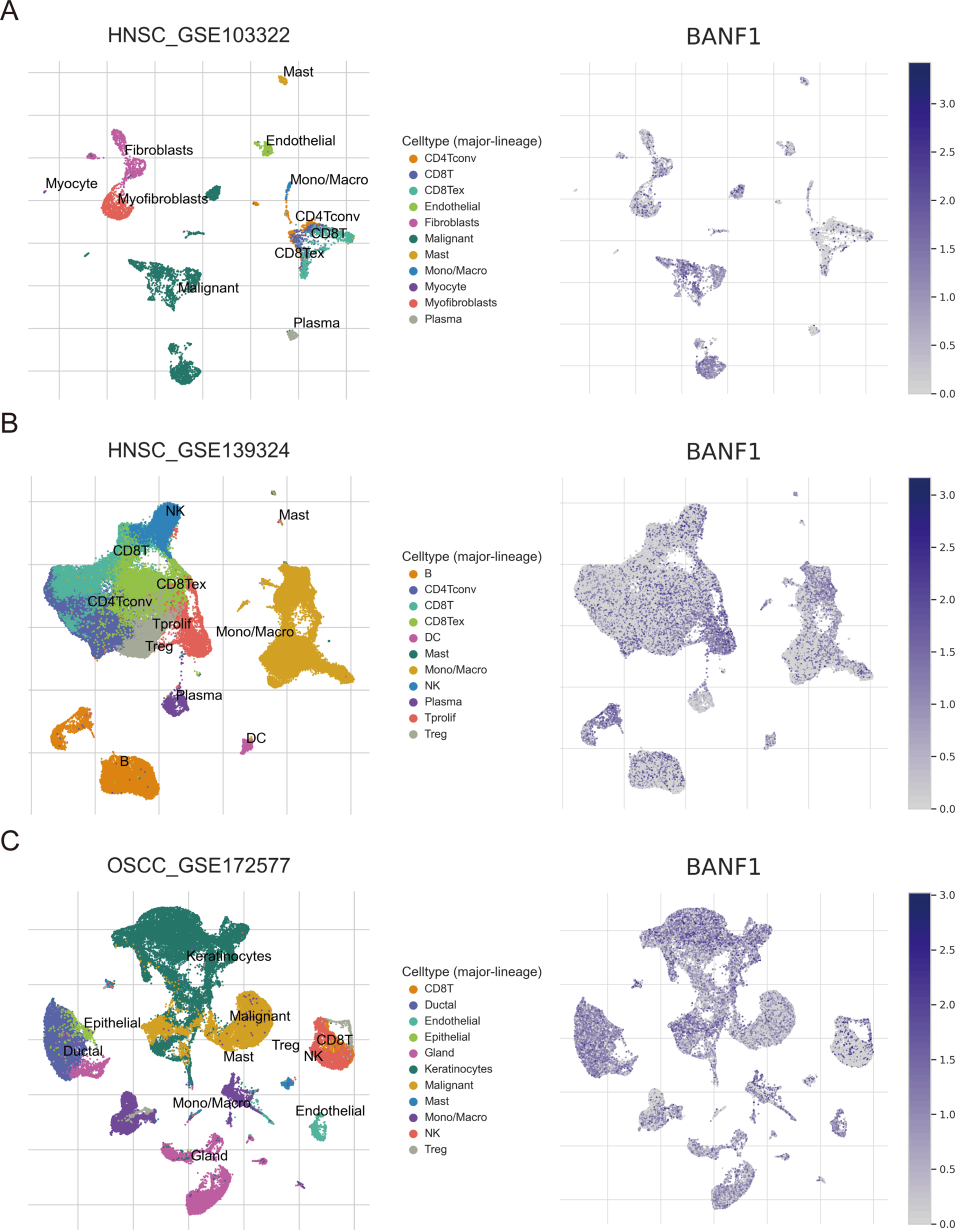
Sequencing analysis of BANF1 single cells in HNSCC from the TISCH website. The main distributions of BANF1 on cell types in **(A)** HNSC_GSE103322, **(B)** HNSC_GSE139324 and **(C)** OSCC_GSE172577 dataset.

**Figure 6:**
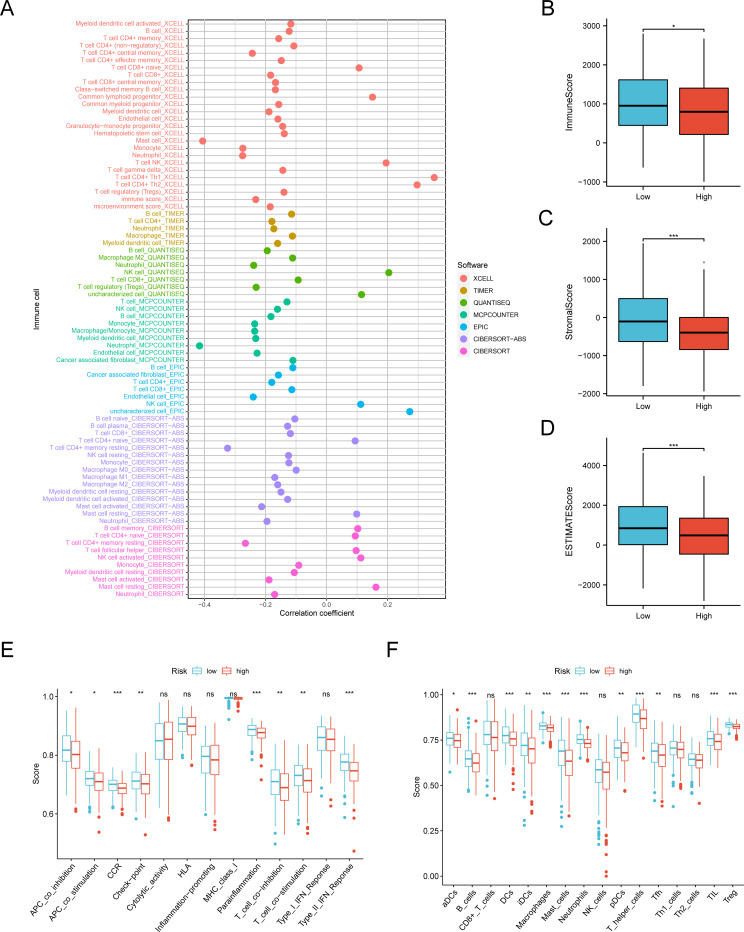
Quantification of immune-invading cells. **(A)** The expression of BANF1 showed a substantial negative correlation with the majority of immune cells. **(B–F)** The BANF1 group with lower levels had elevated TME scores and enhanced immunological activity. *P<0.05, **P<0.01, ***P<0.001. ns indicated no significance.

The prognosis of patients with HNSCC is closely related to multiple indicators of the immune system. To investigate the BANF1 expression in cancerous and immune cells within the TME of HNSCC, we examined three single-cell RNA sequencing databases, namely GSE140228, GSE166635, and GSE98638. **Figures 5A–C** shows that BANF1 expression was observed in both malignant cells and immune cells, such as Tprolif cells, CD8+ T cells, Tregs cells, B cells, macrophages, NK cells, and DC cells. The extensive presence of BANF1 in many types of immune cells provides evidence supporting its potential role in the TME of HNSCC. We evaluated immune cell infiltration in patients with HNSCC to clarify the influence of BANF1 on the TME (**Supplementary Table 2**). A significant negative correlation was found between BANF1 expression and many immune cells, such as Treg cells, mast cells, macrophages, and neutrophils (**Figure 6A**). Furthermore, patients with low expression of BANF1 had higher Estimate, Immune, and Stromal scores compared to the high expression group (**Figures 6B–D**). ssGSEA analysis further suggested that patients with low expression of BANF1 may have more active immune responses (**Figures 6E, F**). These findings confirm our speculation that prolongation of BANF1-associated tumors is strongly associated with immune cell infiltration, which helps to explain the differences in patient survival.

### Prediction of immunotherapy response

**Figure 7:**
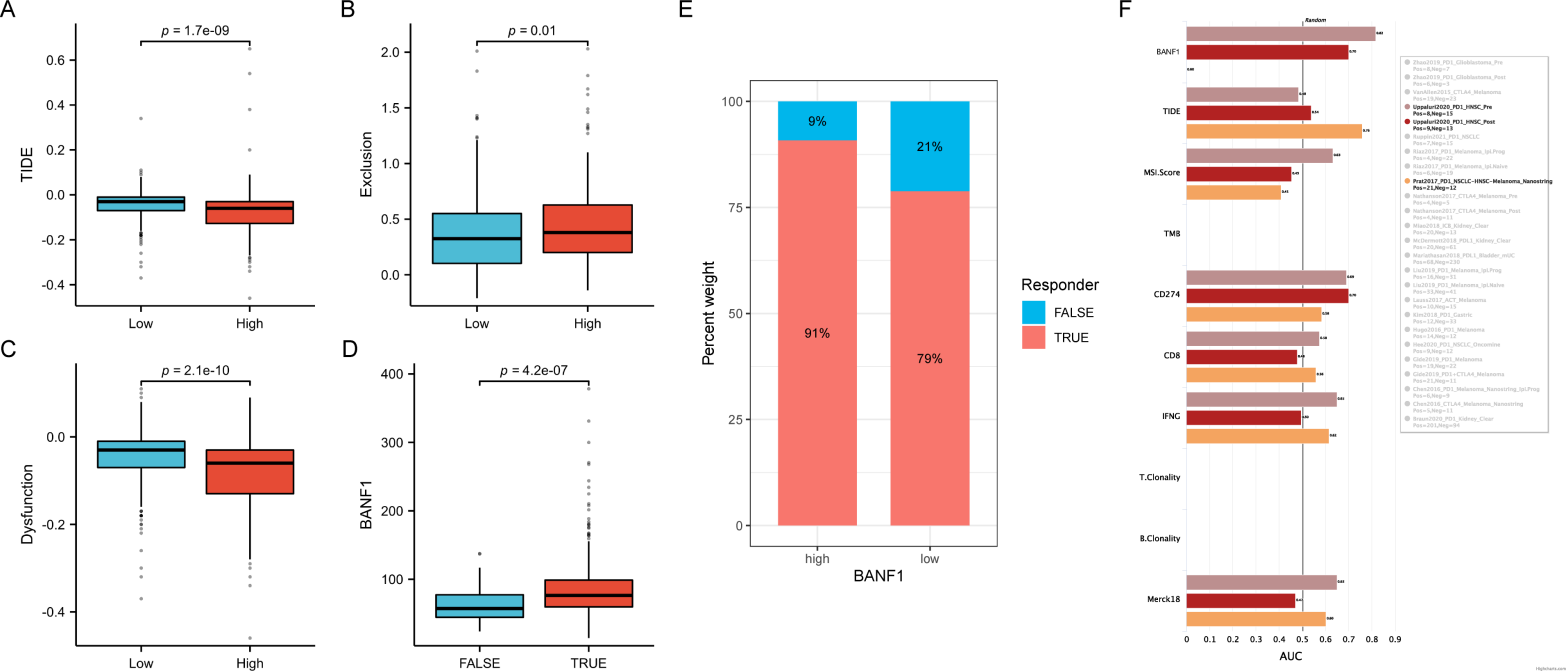
Comparison of immunotherapeutic responses between groups with high and low BANF1 expression. **(A–C)** There are differences in TIDE, Exclusion, and Dysfunction ratings between the groups with high and low expression. **(D)** Differences in BANF1 gene expression between patients with good response to treatment and those with a poor response. **(E)** The proportion of individuals who responded and did not respond in groups with high and low expression in TCGA cohort. **(F)** The TIDE biomarker determines the effectiveness of BANF1 in the immunotherapy response for HNSCC.

We conducted a study using data from the TCGA database to examine the impact of BANF1 expression on immunotherapy responses in patients. The high-expression group exhibited lower TIDE scores, suggesting a more positive response to immunotherapy (**Figure 7A**). The group with low BANF1 expression had higher dysfunction scores, but lower exclusion scores compared to the group with high BANF1 expression (**Figures 7B, C**). Furthermore, our analysis revealed that individuals who responded to treatment exhibited markedly higher levels of BANF1 expression compared with those who did not respond (**Figure 7D**). Furthermore, the findings of the TIDE analysis demonstrated that patients with high expression of BANF1 exhibited a more positive response to ICI treatment compared to those in the low expression group, with response rates of 91% and 79%, respectively (**Figure 7E**). The predictive capacity of BANF1 to determine immunotherapy response was evaluated by evaluating the AUC and comparing them with other established immunotherapy biomarkers such as TIDE, MSI score, Merck18, IFGN, CD8, and CD274 expression. In the cohort of patients with HNSCC who received pretreatment with PD-1, BANF1 showed greater predictive performance compared to CD274. BANF1 had AUC values >0.8, indicating a high probability of a good response to immunotherapy. Furthermore, in the cohort of patients with HNSCC who received PD-1 treatment, the ability of BANF1 to predict results was similar to that of other biomarkers (**Figure 7F**). These findings indicate that BANF1 is a reliable marker for immunotherapy in HNSCC.

### BANF1 and drug response

**Figure 8:**
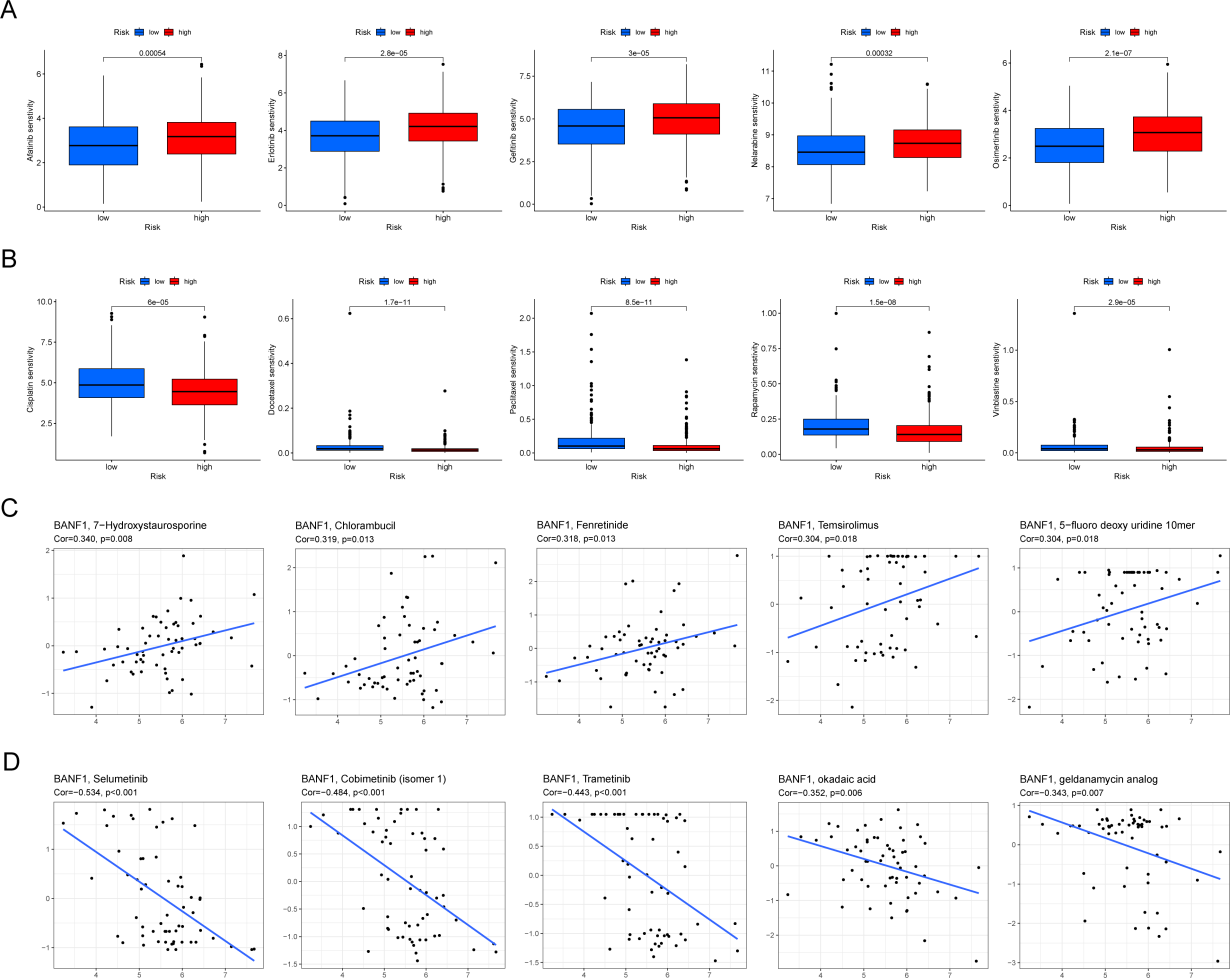
BANF1 is a predictive factor for treatment responsiveness in patients with HNSCC. **(A, B)** Comparative analysis of IC50 values of chemotherapeutic medication in cases of high and low expression of BANF1. **(C, D)** The correlation between BANF1 and HNSCC IC50 values for small and medium-molecule drugs.

We used the “pRRophetic” software tool to analyze the GDSC database and examine the potential to predict the response to chemotherapy in various risk groups by evaluating BANF1 expression. IC50 values of afatinib, erlotinib, gefitinib, and nelarabine were markedly elevated in the high expression group compared to the low expression group, indicating that these treatments would exhibit reduced chemotherapeutic response rates in the high expression group (**Figure 8A**). In contrast, cisplatin, docetaxel, paclitaxel, and rapamycin exhibited lower IC50 values in the high-expression group (**Figure 8B**). This indicates that these medications would be more beneficial for patients with high BANF1 expression levels. There was a favorable correlation between BANF1 expression and response to drugs 7-hydroxystaurosporine, chlorambucil, fenretinide, temsirolimus, and 5-fluoro deoxyuridine 10-mer (**Figure 8C**). Furthermore, a detrimental association was observed between BANF1 expression and the anticancer drugs selumetinib, cobimetinib (isomer 1), trametinib, okadaic acid, and geldanamycin analog (**Figure 8D**). These data provide valuable information to researchers to discover drugs that may exhibit sensitivity or resistance to HNSCC tumors based on their BANF1 expression levels.

### BANF1 promoted HNSCC cell proliferation, migration, and invasion

**Figure 9:**
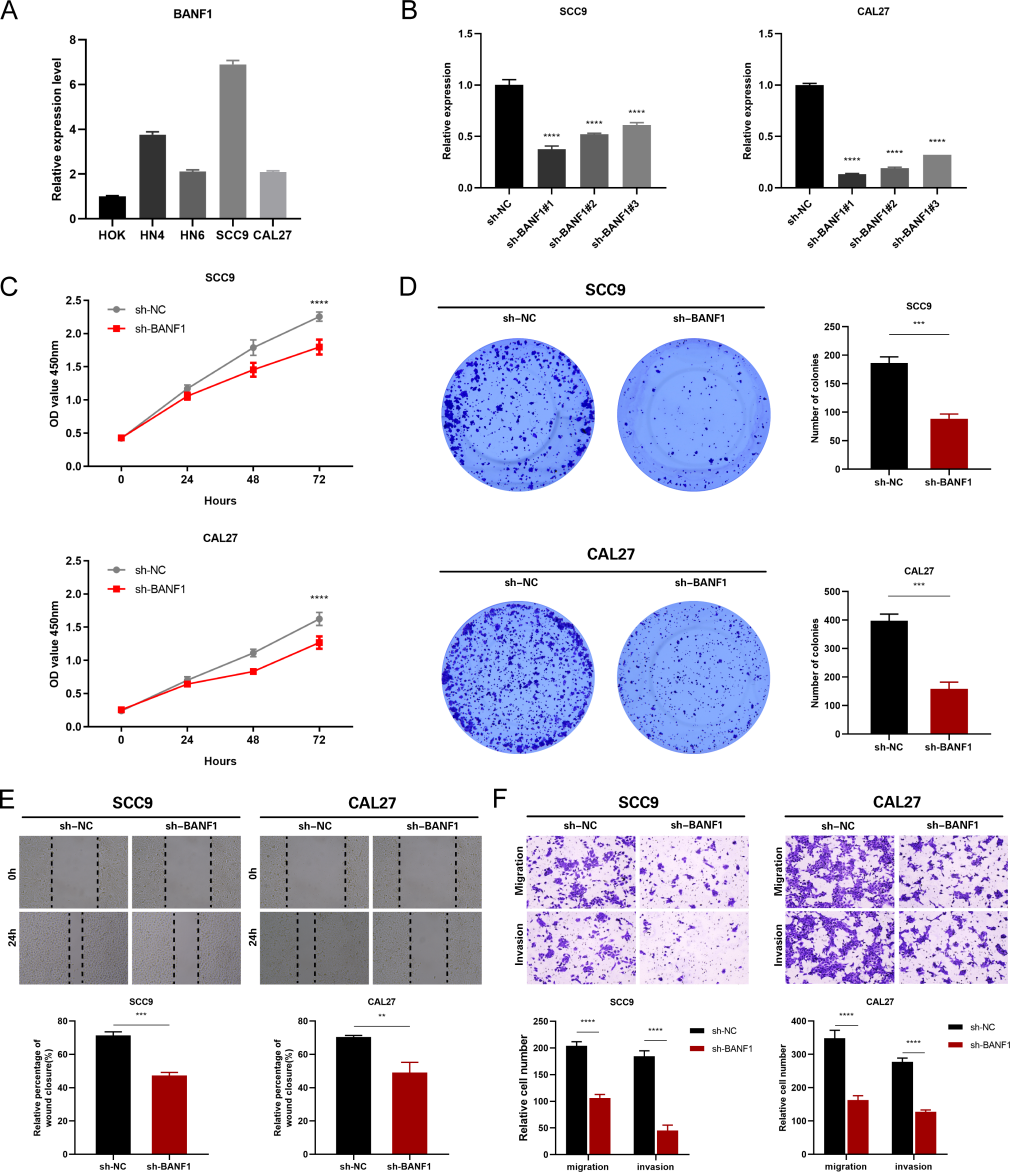
Suppression of BANF1 hindered the growth, movement, and departure of HNSCC cells. **(A)** The qRT-PCR assay successfully identified the presence of BANF1 mRNA expression in HOK, HN4, HN6, SCC9, and CAL27 cells. **(B)** qRT-PCR measured the amounts of BANF1 mRNA in SCC9 and CAL27 cells following transfection. **(C)** Suppression of BANF1 expression hindered the growth and division of HNSCC cells. **(D)** Suppressing the expression of BANF1 hindered the ability of HNSCC cells to generate clones. **(E)** The impact of reduced BANF1 expression on the migratory capacity of HNSCC cells was assessed using a scratch assay. **(F)** The impact of reduced BANF1 expression on the migratory and invasive capabilities of HNSCC cells was assessed using a transwell assay. **P<0.01, ***P<0.001, ****P<0.0001.

**Figure 10:**
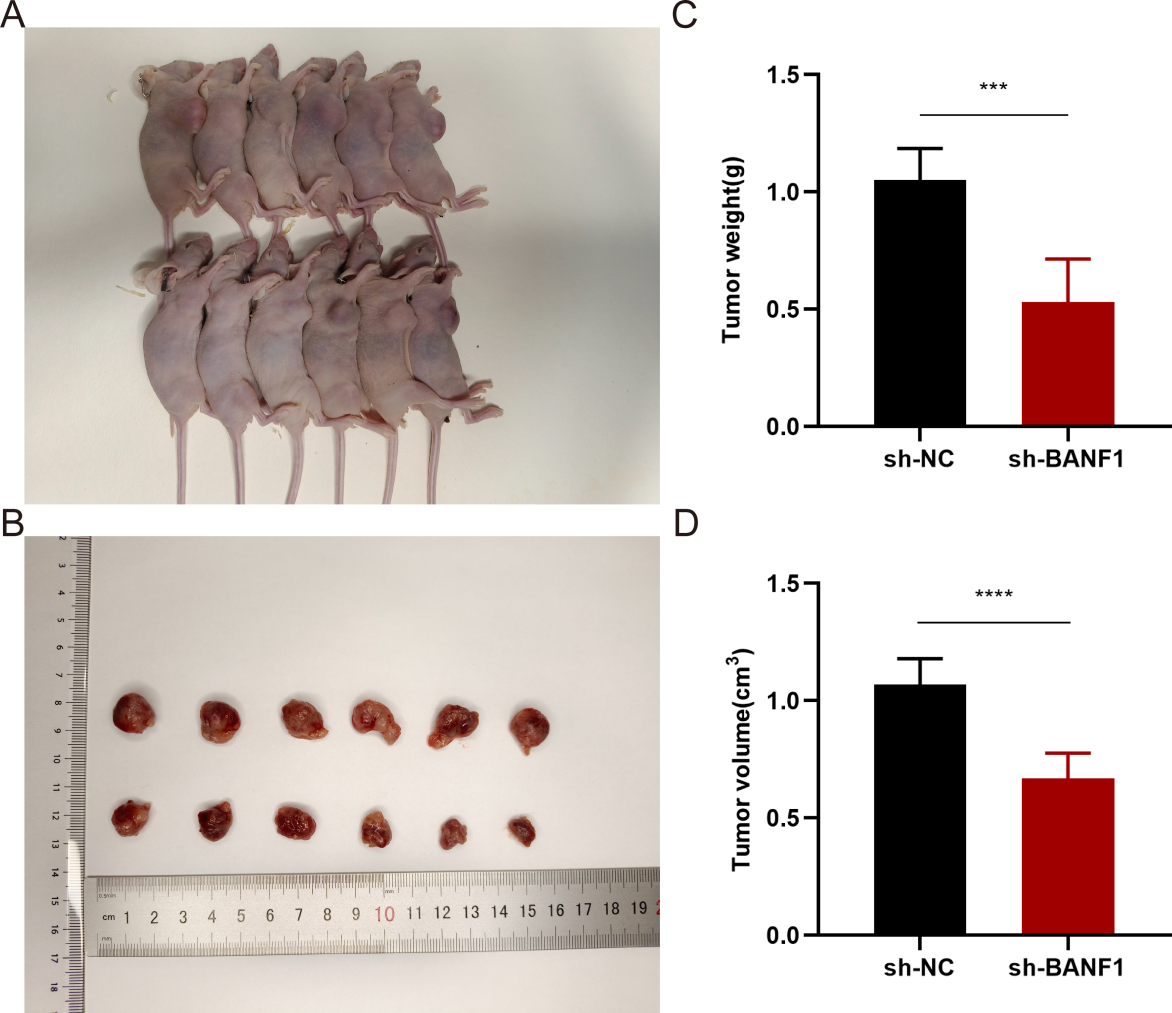
Downregulation of BANF1 expression markedly suppressed the proliferation and expansion of SCC9 cells in a xenograft mice model. **(A)** Tumor formation under the skin in nude mice with varying levels of BANF1 expression. **(B)** Comparative analysis of tumor dimensions. **(C, D)** Comparative analysis of the alterations in weight and volume of subcutaneous tumors in nude mice belonging to distinct BANF1 expression cohorts. ***P<0.001, ****P<0.0001.

To confirm the cancer-causing role of BANF1 in HNSCC, we initially examined BANF1 mRNA expression. Five HNSCC cell lines exhibited higher level of BANF1 expression compared to HOK cells (**Figure 9A**). We created models to silence the BANF1 gene by introducing sh-NC and sh-BANF1 into HNSCC cells by transfection. Following qRT-PCR experiments, the creation of these models was validated in SCC9 and CAL27 cell lines (**Figure 9B**). The CCK-8 assay demonstrated that BANF1 facilitated cell growth (**Figure 9C**). Analysis of colony formation demonstrated that suppression of BANF1 significantly hindered cell cloning capacity in SCC9 and CAL27 cells (**Figure 9D**). We conducted Transwell and scratch assays to examine the impact of BANF1 on HNSCC cell migration and invasion. The wound healing assay demonstrated that inhibition of BANF1 resulted in a decrease in cell migration capacity (**Figure 9E**). Cell migration and invasion were significantly suppressed by silencing BANF1 expression in the Transwell experiment (**Figure 9F**). Furthermore, in conjunction with *in vitro* tests, we created a xenograft HNSCC model in nude mice to investigate the impact of BANF1 on the evolution of HNSCC in a living organism. Subcutaneous injection of BANF1-stabilized and BANF1 knockdown cells SCC9 cells were used to evaluate tumor formation in nude mice. Tumors were collected four weeks after injection. **Figures 10A–D** shows that the tumor volume and weight of the BANF1 down-regulated group were markedly less compared with the control group. This confirms the role of BANF1 in promoting tumor growth in HNSCC.

## Discussion

HNSCC originates in the mucosal epithelium that lines the oral cavity, throat, and larynx (32). HNSCC leads to approximately 550,000 new cases and more than 380,000 deaths worldwide each year (33). The conventional approach to the management of HNSCC involves prioritizing surgery as the primary therapeutic modality. Radiotherapy is frequently employed as a non-operative treatment, as is the combination of radiation, chemotherapy, and surgery (34). Despite recent breakthroughs in diagnostic and imaging procedures, survival rates for individuals with HNSCC have remained unchanged (35). This is due to the frequent recurrence of the disease and the high probability of metastases spreading to the lymph nodes of the neck or to distant regions of the body (36). Recently, the use of ICIs that target the PD-1/PD-L1 pathway has improved the prognosis of patients with metastatic HNSCC (37). However, the results are still not as satisfactory as those seen in other types of cancer, such as melanoma and lung cancer. This difference in outcome may be attributed to the different TME of HNSCC (38). Therefore, it is crucial to identify biomarkers that can accurately predict the effectiveness of immunotherapy for HNSCC.

BANF1, a tiny, nonenzymatic protein, is involved in mitosis and the repair of DNA damage (39). BANF1 is a crucial factor for cell growth and may play a role in tumor development and the transition of normal cells into cancerous cells (40). Wang et al. (27) demonstrated that suppressing BANF1 resulted in restructuring the TME triggered by the immune system, leading to an increased presence of CD8+ T cells and a reduced abundance of myeloid-derived suppressor cells. Xu et al. (27) discovered that both in laboratory settings (*in vitro*) and in living organisms (*in vivo*), BANF1 actively facilitated the proliferation of gastric cancer cells. Furthermore, studies have shown that BANF1 can serve as a predictive biomarker for breast cancer and hepatocellular carcinoma (22, 23). Our findings are consistent with earlier studies, indicating that BANF1 is highly expressed in several types of malignancies, including HNSCC. This observation was further validated by analyzing TCGA and GEO databases. Furthermore, qRT-PCR analysis confirmed that BANF1 exhibited up-regulation in HNSCC cells. Additional research is needed to determine whether inhibition of BANF1 can effectively suppress the malignancy of HNSCC cells, in terms of proliferation, migration, and invasion, which are the primary characteristics of cancer cells. The oncogenic effect of BANF1 knockdown was established using wound healing tests, Transwell invasion assays, and tumor formation assays in nude mice. Our findings suggest that BANF1 could be a promising therapeutic target for HNSCC.

Our investigation revealed a strong correlation between BANF1 expression and various clinicopathological characteristics of HNSCC, including tumor grade, stage, TP53 mutational status, and sex. TP53 (p53) is a highly prevalent oncogene in human malignancies (41). P53 proteins play a crucial role in suppressing tumor growth by repairing DNA damage, controlling metabolism, maintaining normal levels of reactive oxygen species (ROS), regulating the expression of non-coding RNAs, and promoting autophagy or iron regulation (42). Li et al. (43) showed that p53-induced activation of damage-regulated autophagy modulator (DRAM) is related to autophagic breakdown of the vaccinia-related kinase 1 (VRK1) protein. Furthermore, VRK1 depletion hindered BANF1 production. Based on this important study, it can be inferred that the regulatory pathway involving VRK1 and BANF1 may be associated with the p53 signaling pathway. Furthermore, findings from TCGA, GEO, and the validation cohort demonstrated that individuals with greater levels of BANF1 in HNSCC had a diminished overall life expectancy compared to those with lower levels of BANF1. Univariate and multivariate Cox analyses provide additional evidence that elevated BANF1 levels may serve as a significant predictor of HNSCC.

Tumor-infiltrating immune cells are a crucial component of the TME and have been shown to have a significant impact on tumor growth and spread (44). A study using immunohistochemistry revealed that tumors invaded by tumor-infiltrating lymphocytes were correlated with improved outcomes in patients with HNSCC (45). We identified the presence of BANF1 in different immunological infiltrates using single-cell sequencing analysis. Our investigation revealed a negative correlation between BANF1 and immune cells such as Treg cells, mast cells, macrophages, and neutrophils. This suggests that BANF1 may be one of the genes that influences the TME of HNSCC. Furthermore, our analysis revealed that high BANF1 expression was associated with lower ImmuneScore, StromalScore, and EstimateScore indices. This suggests that the high BANF1 expression group may be experiencing an immunosuppressed state. Treg cells play a significant role in the management of HNSCC, as they have the ability to effectively protect against malignancy. Treg cells not only modulate lymphocyte function, but also inhibit tumor cell proliferation, acting as a preventive measure against disease (46). Mast cells carry several Toll-like receptors that, upon receiving external signals, secrete inflammatory cytokines such as IL-6 and IL-13. This secretion facilitates the activation of adaptive and innate immune systems (47). The role of tumor-associated macrophages (TAMs) in HNSCC is widely recognized. An increase in the number of TAMs in the TME is associated with the presence of metastatic lymph node disease and more advanced stages of HNSCC (48, 49). Furthermore, a correlation between TAM and the occurrence of epithelial-mesenchymal transition in OSCC has also be described (50). Neutrophils constitute approximately 60% of the total leukocyte population and therefore serve as the initial defense against inflammation and infection. Invasion of several types of cancer cells can result in the development of advanced malignancies and the existence of these cells can impact on the prognosis of advanced cancers (51). These findings indicate that a TME, characterized by elevated levels of BANF1, has strong immunosuppressive characteristics. This creates a microenvironment that facilitates the growth, invasion, and spread of tumors.

Immunotherapy has emerged as a prominent area of research due to its innovative approach to treatment. PD-L1 functions as an immunological checkpoint, inhibiting autoimmunity, and thus preventing the immune system from eliminating cancer cells (52). Consequently, tumor immunotherapy with ICI has emerged as the primary approach to tumor treatment in contemporary times (53). Although there have been significant advances in immunotherapies, there are still obstacles and challenges that hinder their widespread use in the clinical setting. These include limited response rates, the inability to accurately predict clinical effectiveness, and potential side effects such as autoimmune reactions or cytokine release syndromes. In the last 10 years, there have been attempts to improve immunotherapies and discover biomarkers that indicate how well a patient would respond to immune checkpoint blockade (ICB) (54). Although significant advancements have been made in understanding and reversing the signaling pathways associated with T cell fatigue and senescence, there is currently no optimal treatment regimen or single marker that can accurately differentiate between patients who will respond positively to treatment and those who will not. To assess response to treatment, we made predictions on the efficacy of immunotherapy in both the high- and low-expression groups. Our evaluation, using the TIDE approach, showed substantial efficacy of immunotherapy in patients with HNSCC with high expression of BANF1. We evaluated the precision of BANF1 as a predictor of treatment outcome in five cohorts of patients with HNSCC treated with ICB and contrasted it with other established biomarkers related to tumor immune evasion. Our findings indicated that BANF1 reliably predicted a high probability of a positive response to immunotherapy. Agents targeting PD-1/PD-L1 are highly successful in “hot tumors” that are characterized by a high abundance of CD8+ T cells (55). Therefore, by integrating knowledge of the immune microenvironment and TIDE, we postulated that tumors in the BANF1 high expression category predominantly exhibited the characteristics of “hot tumors,” rendering them more receptive to subsequent immunotherapy.

The present investigation had some limitations. The original dataset for the initial analysis was relatively insufficient, as it was only downloaded from TCGA, prior to drawing any conclusions, it is imperative to verify the predictive significance of BANF1 in an actual clinical population. The database utilized in this study did not include any post-translational changes, thus limiting its ability to comprehensively evaluate the impact of these modifications on BANF1 function. Furthermore, BANF1 overexpression offers only indirect evidence, rather than direct evidence, of alterations in the TME. The association between BANF1 and TME is ambiguous. Further research on the BANF1 response to immunotherapy requires extensive protein sequencing or immunohistochemistry to evaluate and validate the correlation using preclinical and functional studies. Greater efforts are needed to improve clinical efficiency before it can be considered viable treatment options. Although we conducted ex vivo and *in vivo* experiments to demonstrate the tumor-promoting effects of BANF1 in HNSCC, additional trials are required to fully elucidate the role and mechanism of BANF1 in HNSCC.

## Conclusion

Using bioinformatics analysis and conducting studies both *in vitro* and *in vivo*, we investigated the expression level of BANF1 in patients with HNSCC. This study is the first to identify the possible role and prognostic importance of BANF1 in these patients. The findings suggest that elevated expression of BANF1 is an independent predictor of poor prognosis in patients with HNSCC. BANF1 is a compact and versatile protein that is ubiquitous in many cell types and performs a variety of functions. It can also impact the growth, spread, and movement of HNSCC, and it can also play a crucial role in the microenvironment of HNSCC through the infiltration of immune cells. Our data emphasize the potential of BANF1 in predicting ICI efficacy. Finally, we screened drugs associated with BANF1 sensitivity, providing new insights into targeted therapy for HNSCC. BANF1 has been identified as a potentially prognostic biomarker that can serve as both a reliable clinical diagnostic and therapeutic tool, as well as a valuable resource for researchers in developing effective immunotherapy techniques.

## Data Availability

The original contributions presented in the study are included in the article/**Supplementary Material**, further inquiries can be directed to the corresponding author/s.

## References

[1] Cao H Lan T Kuang S Wang L Li J Li Q. FAT1 as a tumor mutation burden specific gene affects the immunotherapy effect in head and neck squamous cell cancer. Drug Resist Update. (2024) 76:101095. doi: 10.1016/j.drup.2024.101095 38986165

[2] Constantin M Chifiriuc MC Bleotu C Vrancianu CO Cristian RE Bertesteanu SV. Molecular pathways and targeted therapies in head and neck cancers pathogenesis. Front Oncol. (2024) 14:1373821. doi: 10.3389/fonc.2024.1373821 38952548 PMC11215092

[3] Gao J Tseng CC Barinsky GL Fang CH Hsueh WD Grube JG. Factors associated with postoperative radiotherapy at a different facility in sinonasal squamous cell carcinoma. Int Forum Allergy Rhinol. (2022) 12:1204–7. doi: 10.1002/alr.22969 34997951

[4] Gupta AK Kanaan M Siddiqi K Sinha DN Mehrotra R. Oral cancer risk assessment for different types of smokeless tobacco products sold worldwide: A review of reviews and meta-analyses. Cancer Prev Res (Phila). (2022) 15:733–46. doi: 10.1158/1940-6207.CAPR-21-0567 36095092

[5] Lan R Campana F Tardivo D Catherine JH Vergnes JN Hadj-Said M. Relationship between internet research data of oral neoplasms and public health programs in the European Union. BMC Oral Health. (2021) 21:648. doi: 10.1186/s12903-021-02022-z 34920710 PMC8679572

[6] Liu W Ding Z Tao Y Liu S Jiang M Yi F. A positive feedback loop between PFKP and c-Myc drives head and neck squamous cell carcinoma progression. Mol Cancer. (2024) 23:141. doi: 10.1186/s12943-024-02051-6 38982480 PMC11232239

[7] Bhat AA Yousuf P Wani NA Rizwan A Chauhan SS Siddiqi MA. Tumor microenvironment: an evil nexus promoting aggressive head and neck squamous cell carcinoma and avenue for targeted therapy. Signal Transduct Target Ther. (2021) 6:12. doi: 10.1038/s41392-020-00419-w 33436555 PMC7804459

[8] Wang HC Chan LP Cho SF. Targeting the immune microenvironment in the treatment of head and neck squamous cell carcinoma. Front Oncol. (2019) 9:1084. doi: 10.3389/fonc.2019.01084 31681613 PMC6803444

[9] Canning M Guo G Yu M Myint C Groves MW Byrd JK. Heterogeneity of the head and neck squamous cell carcinoma immune landscape and its impact on immunotherapy. Front Cell Dev Biol. (2019) 7:52. doi: 10.3389/fcell.2019.00052 31024913 PMC6465325

[10] Cohen EE Lamonte SJ Erb NL Beckman KL Sadeghi N Hutcheson KA. American cancer society head and neck cancer survivorship care guideline. CA Cancer J Clin. (2016) 66:203–39. doi: 10.3322/caac.21343 27002678

[11] Vermorken JB Specenier P. Optimal treatment for recurrent/metastatic head and neck cancer. Ann Oncol. (2010) 21 Suppl 7:i252–61. doi: 10.1093/annonc/mdq453 20943624

[12] Zhang X Yang H Lee JJ Kim E Lippman SM Khuri FR. MicroRNA-related genetic variations as predictors for risk of second primary tumor and/or recurrence in patients with early-stage head and neck cancer. Carcinogenesis. (2010) 31:2118–23. doi: 10.1093/carcin/bgq177 PMC3105587 20819778

[13] Bolderson E Burgess JT Li J Gandhi NS Boucher D Croft LV. Barrier-to-autointegration factor 1 (Banf1) regulates poly [ADP-ribose] polymerase 1 (PARP1) activity following oxidative DNA damage. Nat Commun. (2019) 10:5501. doi: 10.1038/s41467-019-13167-5 31796734 PMC6890647

[14] Cox JL Mallanna SK Ormsbee BD Desler M Wiebe MS Rizzino A. Banf1 is required to maintain the self-renewal of both mouse and human embryonic stem cells. J Cell Sci. (2011) 124:2654–65. doi: 10.1242/jcs.083238 PMC3138706 21750191

[15] Ibrahim N Wicklund A Jamin A Wiebe MS. Barrier to autointegration factor (BAF) inhibits vaccinia virus intermediate transcription in the absence of the viral B1 kinase. Virology. (2013) 444:363–73. doi: 10.1016/j.virol.2013.07.002 PMC3755115 23891157

[16] Samwer M Schneider M Hoefler R Schmalhorst PS Jude JG Zuber J. DNA cross-bridging shapes a single nucleus from a set of mitotic chromosomes. Cell. (2017) 170:956–72. doi: 10.1016/j.cell.2017.07.038 PMC5638020 28841419

[17] Young AM Gunn AL Hatch EM. BAF facilitates interphase nuclear membrane repair through recruitment of nuclear transmembrane proteins. Mol Biol Cell. (2020) 31:1551–60. doi: 10.1091/mbc.E20-01-0009 PMC7521799 32459568

[18] Samson C Petitalot A Celli F Herrada I Ropars V Le Du MH. Structural analysis of the ternary complex between lamin A/C, BAF and emerin identifies an interface disrupted in autosomal recessive progeroid diseases. Nucleic Acids Res. (2018) 46:10460–73. doi: 10.1093/nar/gky736 PMC6212729 30137533

[19] Nichols RJ Wiebe MS Traktman P. The vaccinia-related kinases phosphorylate the N' terminus of BAF, regulating its interaction with DNA and its retention in the nucleus. Mol Biol Cell. (2006) 17:2451–64. doi: 10.1091/mbc.e05-12-1179 PMC1446082 16495336

[20] Margalit A Neufeld E Feinstein N Wilson KL Podbilewicz B Gruenbaum Y. Barrier to autointegration factor blocks premature cell fusion and maintains adult muscle integrity in C. elegans. J Cell Biol. (2007) 178:661–73. doi: 10.1083/jcb.200704049 PMC2064472 17698609

[21] Li J Hu B Fang L Gao Y Shi S He H. Barrier-to-autointegration factor 1: A novel biomarker for gastric cancer. Oncol Lett. (2018) 16:6488–94. doi: 10.3892/ol.2018.9432 PMC6202538 30405787

[22] Shen Q Eun JW Lee K Kim HS Yang HD Kim SY. Barrier to autointegration factor 1, procollagen-lysine, 2-oxoglutarate 5-dioxygenase 3, and splicing factor 3b subunit 4 as early-stage cancer decision markers and drivers of hepatocellular carcinoma. Hepatology. (2018) 67:1360–77. doi: 10.1002/hep.29606 29059470

[23] Vishnubalaji R Abdel-Razeq H Gehani S Albagha O Alajez NM. Identification of a gene panel predictive of triple-negative breast cancer response to neoadjuvant chemotherapy employing transcriptomic and functional validation. Int J Mol Sci. (2022) 23:10901. doi: 10.3390/ijms231810901 36142814 PMC9506546

[24] Ren Z Geng J Xiong C Li X Li Y Li J. Downregulation of VRK1 reduces the expression of BANF1 and suppresses the proliferative and migratory activity of esophageal cancer cells. Oncol Lett. (2020) 20:1163–70. doi: 10.3892/ol.2020.11654 PMC7377186 32724356

[25] Mao L Zhang Y Mo W Yu Y Lu H. BANF1 is downregulated by IRF1-regulated microRNA-203 in cervical cancer. PLoS One. (2015) 10:e117035. doi: 10.1371/journal.pone.0117035 PMC4319761 25658920

[26] Kisling SG Atri P Shah A Cox JL Sharma S Smith LM. A novel HOXA10-associated 5-gene-based prognostic signature for stratification of short-term survivors of pancreatic ductal adenocarcinoma. Clin Cancer Res. (2023) 29:3759–70. doi: 10.1158/1078-0432.CCR-23-0825 PMC10529249 37432996

[27] Wang M Huang Y Chen M Wang W Wu F Zhong T. Inhibition of tumor intrinsic BANF1 activates antitumor immune responses via cGAS-STING and enhances the efficacy of PD-1 blockade. J Immunother Cancer. (2023) 11:e007035. doi: 10.1136/jitc-2023-007035 37620043 PMC10450060

[28] Xu Y Wang X Yuan W Zhang L Chen W Hu K. Identification of BANF1 as a novel prognostic biomarker in gastric cancer and validation via *in-vitro* and *in-vivo* experiments. Aging (Albany NY). (2024) 16:1808–28. doi: 10.18632/aging.205461 PMC10866416 38261746

[29] Sandoval GJ Pulice JL Pakula H Schenone M Takeda DY Pop M. Binding of TMPRSS2-ERG to BAF chromatin remodeling complexes mediates prostate oncogenesis. Mol Cell. (2018) 71:554–66. doi: 10.1016/j.molcel.2018.06.040 PMC6140332 30078722

[30] Fu J Li K Zhang W Wan C Zhang J Jiang P. Large-scale public data reuse to model immunotherapy response and resistance. Genome Med. (2020) 12:21. doi: 10.1186/s13073-020-0721-z 32102694 PMC7045518

[31] Maeser D Gruener RF Huang RS. oncoPredict: an R package for predicting in *vivo* or cancer patient drug response and biomarkers from cell line screening data. Brief Bioinform. (2021) 22:bbab260. doi: 10.1093/bib/bbab260 34260682 PMC8574972

[32] Lee RH Johnson DE Grandis JR. To tip or not to tip: A new combination for precision medicine in head and neck cancer. Cancer Res. (2023) 83:3162–4. doi: 10.1158/0008-5472.CAN-23-1858 PMC10733978 37779427

[33] Yang L Lu P Yang X Li K Chen X Qu S. Excavating novel diagnostic and prognostic long non-coding RNAs (lncRNAs) for head and neck squamous cell carcinoma: an integrated bioinformatics analysis of competing endogenous RNAs (ceRNAs) and gene co-expression networks. Bioengineered. (2021) 12:12821–38. doi: 10.1080/21655979.2021.2003925 PMC8810019 34898376

[34] Iancu D Fulga A Vesa D Zenovia A Fulga I Sarbu MI. Metastatic patterns and treatment options for head and neck cutaneous squamous cell carcinoma (Review). Mol Clin Oncol. (2024) 20:40. doi: 10.3892/mco.2024.2739 38756868 PMC11097132

[35] Kuzume A Chi S Yamauchi N Minami Y. Immune-checkpoint blockade therapy in lymphoma. Int J Mol Sci. (2020) 21:5456. doi: 10.3390/ijms21155456 32751706 PMC7432396

[36] Suresh K Naidoo J Lin CT Danoff S. Immune checkpoint immunotherapy for non-small cell lung cancer: benefits and pulmonary toxicities. Chest. (2018) 154:1416–23. doi: 10.1016/j.chest.2018.08.1048 PMC6335259 30189190

[37] Willsmore ZN Coumbe B Crescioli S Reci S Gupta A Harris RJ. Combined anti-PD-1 and anti-CTLA-4 checkpoint blockade: Treatment of melanoma and immune mechanisms of action. Eur J Immunol. (2021) 51:544–56. doi: 10.1002/eji.202048747 33450785

[38] Henriksen A Dyhl-Polk A Chen I Nielsen D. Checkpoint inhibitors in pancreatic cancer. Cancer Treat Rev. (2019) 78:17–30. doi: 10.1016/j.ctrv.2019.06.005 31325788

[39] Zheng R Ghirlando R Lee MS Mizuuchi K Krause M Craigie R. Barrier-to-autointegration factor (BAF) bridges DNA in a discrete, higher-order nucleoprotein complex. Proc Natl Acad Sci U.S.A. (2000) 97:8997–9002. doi: 10.1073/pnas.150240197 10908652 PMC16810

[40] Rose M Burgess JT O'Byrne K Richard DJ Bolderson E. The role of inner nuclear membrane proteins in tumourigenesis and as potential targets for cancer therapy. Cancer Metastasis Rev. (2022) 41:953–63. doi: 10.1007/s10555-022-10065-z PMC9758098 36205821

[41] Gambichler T Ruddel I Hessam S Bechara FG Stockfleth E Schmitz L. Altered epigenetic pathways and cell cycle dysregulation in healthy appearing skin of patients with koebnerized squamous cell carcinomas following skin surgery. J Eur Acad Dermatol Venereol. (2018) 32:1485–91. doi: 10.1111/jdv.14887 29478287

[42] Duffy MJ Synnott NC Crown J. Mutant p53 as a target for cancer treatment. Eur J Cancer. (2017) 83:258–65. doi: 10.1016/j.ejca.2017.06.023 28756138

[43] Li J Wang T Pei L Jing J Hu W Sun T. Expression of VRK1 and the downstream gene BANF1 in esophageal cancer. BioMed Pharmacother. (2017) 89:1086–91. doi: 10.1016/j.biopha.2017.02.095 28298069

[44] Padoan A Plebani M Basso D. Inflammation and pancreatic cancer: focus on metabolism, cytokines, and immunity. Int J Mol Sci. (2019) 20:676. doi: 10.3390/ijms20030676 30764482 PMC6387440

[45] Peltanova B Raudenska M Masarik M. Effect of tumor microenvironment on pathogenesis of the head and neck squamous cell carcinoma: a systematic review. Mol Cancer. (2019) 18:63. doi: 10.1186/s12943-019-0983-5 30927923 PMC6441173

[46] Economopoulou P Agelaki S Perisanidis C Giotakis EI Psyrri A. The promise of immunotherapy in head and neck squamous cell carcinoma. Ann Oncol. (2016) 27:1675–85. doi: 10.1093/annonc/mdw226 27380958

[47] Senovilla L Vacchelli E Galon J Adjemian S Eggermont A Fridman WH. Trial watch: Prognostic and predictive value of the immune infiltrate in cancer. Oncoimmunology. (2012) 1:1323–43. doi: 10.4161/onci.22009 PMC3518505 23243596

[48] Kumar AT Knops A Swendseid B Martinez-Outschoom U Harshyne L Philp N. Prognostic significance of tumor-associated macrophage content in head and neck squamous cell carcinoma: A meta-analysis. Front Oncol. (2019) 9:656. doi: 10.3389/fonc.2019.00656 31396482 PMC6663973

[49] Yang L Zhang Y. Tumor-associated macrophages: from basic research to clinical application. J Hematol Oncol. (2017) 10:58. doi: 10.1186/s13045-017-0430-2 28241846 PMC5329931

[50] Gao L Zhang W Zhong WQ Liu ZJ Li HM Yu ZL. Tumor associated macrophages induce epithelial to mesenchymal transition via the EGFR/ERK1/2 pathway in head and neck squamous cell carcinoma. Oncol Rep. (2018) 40:2558–72. doi: 10.3892/or.2018.6657 PMC6151899 30132555

[51] Donskov F. Immunomonitoring and prognostic relevance of neutrophils in clinical trials. Semin Cancer Biol. (2013) 23:200–7. doi: 10.1016/j.semcancer.2013.02.001 23403174

[52] Kolb HJ Mittermuller J Clemm C Holler E Ledderose G Brehm G. Donor leukocyte transfusions for treatment of recurrent chronic myelogenous leukemia in marrow transplant patients. Blood. (1990) 76:2462–5. doi: 10.1182/blood.V76.12.2462.2462 2265242

[53] Rocken M. Early tumor dissemination, but late metastasis: insights into tumor dormancy. J Clin Invest. (2010) 120:1800–3. doi: 10.1172/JCI43424 PMC2877965 20501952

[54] Waldman AD Fritz JM Lenardo MJ. A guide to cancer immunotherapy: from T cell basic science to clinical practice. Nat Rev Immunol. (2020) 20:651–68. doi: 10.1038/s41577-020-0306-5 PMC7238960 32433532

[55] Galon J Bruni D. Approaches to treat immune hot, altered and cold tumours with combination immunotherapies. Nat Rev Drug Discovery. (2019) 18:197–218. doi: 10.1038/s41573-018-0007-y 30610226

[56] Kobayashi S Koujin T Kojidani T Osakada H Mori C Hiraoka Y. BAF is a cytosolic DNA sensor that leads to exogenous DNA avoiding autophagy. Proc Natl Acad Sci U.S.A. (2015) 112:7027–32. doi: 10.1073/pnas.1501235112 PMC4460496 25991860

[57] Zhang G. Expression and prognostic significance of BANF1 in triple-negative breast cancer. Cancer Manag Res. (2020) 12:145–50. doi: 10.2147/CMAR.S229022 PMC6955598 32021431

